# Frailty syndrome in an independent urban population in Brazil (FIBRA study): a cross-sectional populational study: Síndrome da fragilidade em uma população urbana independente no Brasil (estudo FIBRA): um estudo transversal populacional

**DOI:** 10.1590/1516-3180.2016.0078180516

**Published:** 2016-09-19

**Authors:** Larissa Barradas Calado, Eduardo Ferriolli, Júlio César Moriguti, Edson Zangiacomi Martinez, Nereida Kilza da Costa Lima

**Affiliations:** I MD, MSc. Student in the Postgraduate Program on Internal Medicine, Department of Internal Medicine, Faculdade de Medicina de Ribeirão Preto (FMRP), Universidade de São Paulo (USP), Ribeirão Preto, SP, Brazil.; II MD, PhD. Associate Professor, Division of General Internal Medicine and Geriatrics, Department of Internal Medicine, Faculdade de Medicina de Ribeirão Preto (FMRP), Universidade de São Paulo (USP), Ribeirão Preto, SP, Brazil.; III MD, PhD. Associate Professor, Department of Social Medicine, Faculdade de Medicina de Ribeirão Preto (FMRP), Universidade de São Paulo (USP), Ribeirão Preto, SP, Brazil.

**Keywords:** Frail elderly, Socioeconomic factors, Morbidity, Cross-sectional studies, Urban population, Idoso fragilizado, Fatores socioeconômicos, Morbidade, Estudos transversais, População urbana

## Abstract

**CONTEXT AND OBJECTIVE:**

Frailty is a multifactorial syndrome. The aim of this study was to determine the prevalence and characteristics of frailty syndrome in an elderly urban population.

**DESIGN AND SETTING:**

Cross-sectional study carried out at the homes of a randomized sample representing the independent elderly individuals of Ribeirão Preto, Brazil.

**METHODS:**

Sociodemographic characteristics, clinical data and criteria of the frailty phenotype were obtained at the subjects’ homes; 385 individuals were evaluated. Frailty was defined based on detection of weight loss, exhaustion, weakness, slowness and low physical activity level. Individuals with three or more of these characteristics were classified as frail and those with one or two as pre-frail. Specific cutoff points for weakness, slowness and low physical activity level were calculated.

**RESULTS:**

The participants’ mean age was 73.9 ± 6.5 years, and 64.7% were women. 12.5% had lost weight over the last year; 20.5% showed exhaustion, 17.1% slowness, 24.4% low physical activity level and 20.5% weakness. 9.1% were considered frail and 49.6% pre-frail. Frail subjects were older, attended more medical visits, had a higher chance of hospitalization within the last 12 months and had more cerebrovascular events, diabetes, neoplasms, osteoporosis and urinary and fecal incontinence.

**CONCLUSION:**

In this independent elderly population, there were numerous frail and pre-frail individuals. Frailty syndrome was associated with high morbidity. Cutoff points for weakness, slowness and low physical activity level should be adjusted for the population under study. It is essential to identify frail and pre-frail older individuals for appropriate interventions.

## RESUMO

**CONTEXTO E OBJETIVO:**

Fragilidade é uma síndrome multifatorial. O objetivo deste estudo foi determinar a prevalência e características da síndrome da fragilidade em uma população urbana de idosos.

**TIPO DE ESTUDO E LOCAL:**

Estudo transversal realizado nas casas em amostra randomizada para representar os idosos independentes de Ribeirão Preto, Brasil.

**MÉTODO:**

Características sociodemográficas, dados clínicos e critérios do fenótipo da fragilidade foram obtidos nas casas. Foram avaliados 385 idosos. A definição da fragilidade foi baseada na detecção de perda de peso, exaustão, fraqueza, lentidão e baixo nível de atividade física. Idosos com três ou mais destas características foram classificados com frágeis; com uma ou duas características como pré-frágeis. Foram calculados pontos de corte específicos para fraqueza, lentidão e baixo nível de atividade física.

**RESULTADOS:**

A média de idade dos participantes foi de 73,9 ± 6,5 anos, com 64,7% de mulheres. 12,5 % perderam peso no último ano, 20,5% tiveram exaustão, 17,1% lentidão, 24,5% baixo nível de atividade física e 20,5% fraqueza. Foram considerados frágeis 9,1% e pré-frágeis 49,6%. Os frágeis eram mais velhos, foram em mais consultas médicas, tiveram maior chance de internação nos últimos 12 meses e tiveram mais eventos cerebrovasculares, diabetes, neoplasias, osteoporose, incontinência fecal e urinária.

**CONCLUSÃO:**

Em uma população idosa independente, existem vários indivíduos frágeis e pré-frágeis. A síndrome da fragilidade foi associada com alta morbidade. Pontos de corte para fraqueza, lentidão e baixo nível de atividade física devem ser ajustados para a população em estudo. É essencial identificar idosos frágeis e pré-frágeis para intervenções apropriadas.

## INTRODUCTION

Frailty is a clinical multifactorial syndrome involving reduction of the functional reserves and dysfunction of various organic systems. It results in marked reduction of the ability to reestablish functions after aggression of various natures.^1^

Based on this multifactorial concept, Fried et al. proposed that a phenotype with five components should be envisaged: weight loss, exhaustion, weakness, slowness and low physical activity.^2^ Individuals with three or more of these characteristics would be classified as frail and those with one or two characteristics as pre-frail. According to Fried et al., older individuals who meet these criteria are more susceptible to falls, functional decline, recurrent hospitalization and death within three years.^2^ These criteria are the ones most frequently used by investigators in epidemiological and clinical studies.^3^^,^^4^^,^^5^

The incidence and prevalence of frailty syndrome vary and are influenced by the geographic location studied, by socioeconomic factors such as education, and by age, such that its incidence and prevalence are higher among individuals aged 80 years or over.^2^^,^^3^^,^^6^^,^^7^^,^^8^^,^^9^^,^^10^

Studies conducted both in Brazil and in other countries have reported different prevalence rates, ranging from 6.9% to 40.6% among frail elderly individuals and 46.3% to 60.1% among pre-frail individuals.^2^^,^^4^^,^^11^^,^^12^^,^^13^^,^^14^ In this regard, frailty syndrome should be targeted by earlier investigations and interventions, given its impact on elderly individuals, their families and society as a whole.^1^ Despite recent initiatives, there are few Brazilian studies assessing this condition, and interventions that reduce the impact of this syndrome are still insufficient.^15^

## OBJECTIVE

The aims of this study were to determine the prevalence and sociodemographic and clinical profiles of frailty syndrome in an independent elderly urban Brazilian population, and to calculate specific cutoff points for weakness, slowness and low physical activity for populations similar to the one studied here. These parameters are influenced by anthropometric data and they are reflected in the prevalence of frailty syndrome.

## METHODS

### Setting and participants

A population-based epidemiological cross-sectional study, forming part of the multicenter “Study of Frailty in Elderly Brazilian Individuals” (FIBRA), was conducted in Ribeirão Preto, state of São Paulo, Brazil. This city has approximately 600,000 inhabitants, 8.7% of them over 65 years of age (Brazilian Institute for Geography and Statistics).

The inclusion criteria were that the subjects needed to be aged 65 years or over and living in a randomly selected house. The sample was obtained in a random manner from area conglomerates and its size was calculated by estimating the proportion of frailty (i.e. the value at which the sample size obtained would be the maximum possible: P = 0.50; q = 0.50), according to the older population of the city. The significance level was set at 5% (alpha = 5%; Z = 1.96). The sample size was established as 385 volunteers for a sampling error of 5%.

The exclusion criteria comprised presence of severe cognitive deficit suggestive of dementia after evaluation by the Mini Mental State Examination (MMSE); situations of being bedridden or needing to use a wheelchair; presence of sequelae from a stroke with localized loss of strength; greater age with presentation of end-stage disease; or situations of undergoing treatment for cancer, except for skin cancer.

In order to obtain a representative sample of the elderly population of Ribeirão Preto, we calculated the proportion of older individuals in each neighborhood in relation to the total number of older individuals in the city, on the basis of data from the Brazilian Institute for Geography and Statistics. We then calculated the density of elderly people per household in each neighborhood. The city blocks in which households were visited were identified and selected randomly using maps supplied by the Brazilian Institute for Geography and Statistics and by the city’s administration. If the home was inhabited by more than one elderly individual, all of them were invited to participate. Situations in which the elderly individuals categorically stated that they did not want to participate or that they wanted to discontinue participation, after starting, were considered to be refusals. When an elderly individual was present and interested in participating, but declared that he or she was unable to do so at that moment, or a resident or neighbor said that an elderly person lived in the house but was not present at that moment, the interviewer scheduled a new visit, orally and in writing, up to a total of three attempts. If the house was closed on the occasion of the first visit by the interviewer, and it was not possible to know whether an elderly individual lived there, this was considered to be a loss of data. Refusal did not involve exclusion of the home from the total count because the sample calculation had taken the possibility of this occurrence into consideration.

An additional proportional number of city blocks was selected in each neighborhood in order to cover for absences and to replace losses due to the exclusion criteria of the study. The elderly individuals were invited to participate through personal contact at their homes.

### Measurements

A standard assessment tool was formulated and first tested in a pilot study in order to check for any failures and to obtain improvements during the formulation process. The pilot study was conducted in Ribeirão Preto in 2008/2009. It involved undergraduate and postgraduate students at the Ribeirão Preto Medical School and Ribeirão Preto College of Nursing, University of São Paulo, and recruited volunteers. All of this investigative team received prior training at four meetings. Kits containing a tape measure, scale, caliper, a dynamometer for measuring handgrip strength and markers for the walking test were acquired for applying the protocol after the pilot study had been concluded.

In the standard questionnaire, the subjects were asked to self-report any chronic diseases that had been recognized by a doctor during the past year, and also to state the number of visits to doctors and admissions to hospital that they had had, also during the past year.

The frailty criteria adopted in the present study were based on the clinical syndrome described and defined by Fried et al.^2^ Their definition originated within the context of cardiovascular health studies and women’s health and aging studies. The group proposed that a frailty phenotype with five components should be envisaged, defined operationally as described below. These criteria have now become established and are widely used in studies on frailty.

1. Weight loss: report of a loss ≥ 4.5 kg or ≥ 5% of body weight during the previous year.
2. Exhaustion: assessed through a self-report of fatigue in response to two questions on the Center for Epidemiological Studies Depression Scale (CES-D). When a subject stated that on three or more days of the week he felt he had to make much more effort to carry out his usual tasks and could not continue them, he received scores for fatigue.
3. Weakness: mean of three measurements with a hydraulic dynamometer (JAMAR Model J00105) on the dominant hand, adjusted for sex and body mass index (BMI). Subjects were considered to be positive when their handgrip strength was below the 20^th^ percentile for this population.^2^
4. Low physical activity level: measured from the weekly energy expenditure in kilocalories, with adjustment for sex, based on self-reported activities and physical exercise, and assessed using the Minnesota Leisure Time Activities Questionnaire, which has been validated in Brazil.^16^ Subjects in the lowest quartile were considered to be positive for this criterion.^2^
5. Slowness: measured from the time, in seconds, taken to walk 4.6 m and adjusted for sex and height. This criterion was positive for the slowest 20% of the subjects.^2^

Older subjects with three or more of these characteristics were classified as frail and those with one or two characteristics as pre-frail.^2^

The study by Fried et al.^2^ proposed cutoff points for the criteria of weakness, low physical activity and slowness criteria, since calculation of these criteria depends on the anthropometric variables and intrinsic characteristics of each population. However, in this study we chose to calculate specific cutoff points for the sample and compared them with the values obtained by Fried et al.^2^

### Ethics

Our study was approved by the Ethics Committee of the University Hospital, Ribeirão Preto Medical School, University of São Paulo. Written informed consent was obtained from all participants.

### Statistics

Descriptive data were analyzed: means and standard deviations (SD) were used for continuous variables and proportions were calculated for categorical variables. Differences between frail groups were investigated using the Fisher exact test for categorical variables, and analysis of variance (ANOVA) followed by the Bonferroni test was used for continuous and parametric variables. All analyses were carried out using the SAS 9.0 software, and the significance level was set at P < 0.05.

## RESULTS

A total of 385 individuals were studied, among whom 249 (64.7%) were women. The overall mean age of the participants was 73.9 ± 6.5 years, with means of 74.0 ± 6.6 years for men and 73.9 ± 6.5 years for women (P = 0.84). The participants were predominantly white (68.2%), had 1 to 4 years of schooling (54.3%), were married (58.4%) and had four or more children (46.2%) (Table 1).

**Table 1.** Characteristics of the study population (n = 385)

Variable	Men		Women		All	
	n = 136	%	n = 249	%	n = 385	%
Ethnicity						
Caucasians	96	70.6	167	67.1	263	68.2
Others	40	29.4	82	32.9	122	31.7
Schooling (years)						
0	22	16.2	57	22.9	70	20.5
1 to 4	74	54.4	135	54.2	209	54.3
5 to 12	29	21.3	40	16.0	69	17.9
13 or over	11	8.1	17	6.8	28	7.3
Marital status						
Single	7	5.1	19	7.6	26	6.7
Married	110	80.9	115	46.2	225	58.4
Widowed	14	10.3	97	39.0	111	28.8
Divorced	5	3.7	18	7.2	23	6.1
Number of children						
0	12	8.8	27	10.8	39	10.1
1	13	9.6	18	7.2	31	8.0
2 or 3	53	39.0	84	33.8	137	35.6
4 or more	58	42.6	120	48.2	178	46.2
Reported diseases						
Heart disease	17	12.5	31	12.4	48	12.5
Hypertension	60	44.1	118	47.4	178	46.2
Stroke	4	2.9	4	1.6	8	2.1
Diabetes	23	16.9	44	17.7	67	17.4
Cancer	6	4.4	5	2	11	2.9
Arthritis	15	11.0	48	19.3	63	16.4
Depression	13	9.6	60	24.1	73	18.9
Respiratory disease	13	9.6	13	5.2	26	6.7
Osteoporosis	6	4.4	76	30.5	82	21.3
Urinary incontinence	33	24.3	86	34.5	119	30.9
Number of medications						
0	23	16.9	22	8.8	45	11.7
1	32	23.5	34	13.7	66	17.1
2	22	16.2	41	16.5	63	16.4
3	14	10.3	43	17.3	57	14.8
4 or more	45	33.1	108	43.4	154	39.7

In this study population, systemic arterial hypertension was the most common self-reported disease (46.2%), followed by urinary incontinence (30.9%) and osteoporosis (21.3%). More than one third of the participants reported that they took four or more medications per day (Table 1).

According to the frailty criteria used in the present study, 9.1% of the sample was considered frail, 49.6% pre-frail and 41.3% non-frail. The mean age was 72.2 ± 5.9, 74.7 ± 6.4 and 77.7 ± 7.6 years for the non-frail, pre-frail and frail groups, respectively (P < 0.01). Post-test analysis showed that there were differences in age between all groups: frail versus non-frail (P < 0.01), frail versus pre-frail (P = 0.01) and non-frail versus pre-frail (P = 0.01).

Regarding the frailty criteria, 12.5% were positive for weight loss over the last year, 20.5% for exhaustion, 17.1% for slowness, 24.4% for low physical activity and 20.5% for weakness. The exhaustion criterion was mainly reported by females (P < 0.001), whereas no difference between the sexes was observed for the remaining criteria.

The cutoff points for weakness and slowness calculated for our elderly population are shown in Tables 2 and 3. For the criterion of low physical activity, women with a Minnesota value of 0 (zero) kcal/week and men with values below 107.49 kcal/week were scored as frail.

**Table 2.** Reference values for hand grip strength score

BMI (kg/m^2^)	Cutoff (kgf)	
	Men	Women
≤ 23	< 17.33	< 12.87
> 23 to ≤ 28	< 24.93	< 14.27
> 28 to ≤ 30	< 28.27	< 10.53
> 30	< 18	< 16.40

BMI = body mass index; kgf = kilogram force.

**Table 3.** Reference values for slowness score (4.6-m walk test)

Height (cm)	Cutoff (seconds)
Men < 168	> 4.88
Men > 168	> 4.97
Women < 155	> 5.73
Women > 155	> 6.33

Analysis on the associations between the sociodemographic variables listed in Table 1 and frailty did not reveal any differences between the levels of the syndrome (P > 0.05). There were associations between frailty and stroke (P = 0.02), diabetes (< 0.001), neoplasia (P = 0.02), osteoporosis (P = 0.007), urinary incontinence (P = 0.001) and fecal incontinence (P = 0.002). The elderly subjects who were considered to be frail attended more medical visits (P = 0.03) and had a greater chance of having been hospitalized (P < 0.001) within the 12 months preceding the interview than did non-frail and pre-frail subjects. No difference was observed between the frail, pre-frail and non-frail groups regarding the number of medications used (P = 0.54) (Table 4).

**Table 4.** Associations between self-reported health conditions and frailty syndrome

Variable	Non-frail		Pre-frail		Frail		P
	n = 159	%	n = 191	%	n = 35	%	
Heart diseases	15	9.4	24	12.6	9	25.7	0.09
Hypertension	70	44.0	91	47.6	17	48.6	0.81
Stroke	0	0	5	2.6	3	8.6	0.02
Diabetes	15	9.4	38	19.9	14	40.0	< 0.001
Cancer	3	1.9	4	2.1	4	11.4	0.02
Arthritis	22	13.8	32	16.7	9	2.6	0.51
Depression	8	5.0	12	6.3	6	1.7	0.10
Respiratory diseases	27	17.0	34	17.8	12	3.4	0.14
Osteoporosis	26	16.4	41	21.5	15	42.9	0.007
Urinary incontinence	33	20.8	71	37.2	15	42.9	0.001
Fecal incontinence	2	1.3	4	2.1	4	11.4	0.002
Number of medications per day							
0	25	15.7	18	9.4	2	5.7	0.54
1	36	22.7	25	13.1	5	14.3	
2	26	16.3	31	16.2	6	17.2	
3	23	14.5	30	15.7	4	11.4	
4+	49	30.8	87	45.6	18	51.4	
Number of doctor visits over the last 12 months							
0	18	11.3	15	7.9	0	0	0.03
1 to 4	101	63.5	115	60.2	18	51.4	
5 or more	40	25.2	61	31.9	17	48.6	
Hospitalization over the last 12 months							
Yes	22	13.8	37	19.4	15	42.8	< 0.001
No	137	86.2	154	80.6	20	57.2	

## DISCUSSION

In this study, 9.1% of the sample was frail, 49.6% pre-frail and 41.3% non-frail. Data regarding frailty syndrome are necessary for planning public health policies, since this is considered to be a clinical, non-unidirectional and potentially reversible syndrome.^17^ Early recognition and adoption of proactive measures at different stages of the process can prevent or delay the occurrence of adverse health outcomes. In Brazil, this information was scarce before the creation of the FIBRA Network project, to which the present study belongs.

The prevalence of the frailty syndrome differed according to the region studied. In Santa Cruz, a city in the state of Rio Grande do Norte, the prevalence of frailty was found to be 17.1%. This value is higher than those found in developed countries, perhaps as a result of exposure to various adverse factors and stressors that typically affect people’s lives in that region.^4^ The SABE 2000 study, conducted in the city of São Paulo, evaluated 688 elderly individuals aged 75 years or over and found that the prevalence of frailty was 29.5% and pre-frailty, 50.6%.^18^ The large number of frail subjects in that study was due, in part, to the greater age of the sample. Moreover, the prevalence of frailty was 9% in Campinas, state of São Paulo, a city with 1.1 million inhabitants,^13^ and 8.7% in Belo Horizonte, state of Minas Gerais state,^14^ which has around 2.3 million. These rates were similar to those detected in the present study. All of these cities have high human development indexes (HDI).^13^^,^^14^^,^^18^

The prevalence of the components of frailty syndrome tends to be higher in places with a low HDI.^13^ The percentage of frailty syndrome detected in the population of the present study was similar to that observed in developed countries.^2^^,^^3^ This may partly be explained by the high HDI of the city of Ribeirão Preto, which is considered to be one of the highest in the state of São Paulo and in Brazil, and close to what is seen in developed countries.^2^^,^^3^ In contrast, in places where the HDI is considered to be medium or low, there is a high prevalence of frailty. Sousa et al. believed that prevalences of frailty higher than those found in developed countries may be due to lifelong exposure to different adverse and stressful factors that are characteristic of vulnerable regions.^4^ Factors such as precarious social conditions, childhood lived in poverty, adverse working conditions during adulthood, situations of health risk and violence may indirectly interfere with the development of subclinical inflammatory processes and with the immune response to stress. These processes are closely linked to frailty^4^.

Frailty was significantly associated with age. Many studies have shown the influence of aging on the process of becoming frail.^2^^,^^19^^,^^20^^,^^21^ Advancing age is a complex phenomenon that it is difficult to classify into different levels. It is associated with progressive loss of homeostatic and hemodynamic regulation, which makes the body less resilient, and often not at all resilient, to adverse situations, depending on the functional reserves of different physiological systems. It is in this context that the frailty syndrome arises.^22^ Thus, implementation of diagnostic efforts and early intervention regarding the consequences of normal and pathological decline due to aging is unquestionably an important public health issue.

No other association between frailty and sociodemographic variables was detected in the present study, probably because there was some homogeneity of schooling and family characteristics among the older subjects of this population. Woo et al. observed that the frailty index was higher among subjects of low educational level, with low income and with inadequate financial situations.^23^ Espinoza et al. observed an association between low schooling levels and the onset of new cases of frailty, while Bilotta et al. reported that the syndrome was associated with age, female sex, widowhood, dependence on other people for activities of daily living, depression, comorbidities and use of several medications.^6^^,^^9^

In the present study, only 7% of the subjects who were considered to be frail did not have any chronic disease, and 25% of this group reported having hypertension. The frail group attended more medical visits than the non-frail group. Associations with stroke, diabetes, neoplasia and urinary and fecal incontinence were also observed. Individuals presenting frailty syndrome have lower capacity to respond to adverse events, possibly due to reduced functional reserves. Frail elderly people are recognized to be at high risk of adverse health results and to have impaired ability to tolerate hospitalization and invasive procedures. Thus, this group has accentuated needs for health services, care and institutionalization, as well as high mortality.^24^ Furthermore, with aging, the number of visits to doctors increases considerably. People aged 75 years or over attend around seven visits to doctors per year for monitoring of both acute and chronic illnesses.^25^

The relationship between frailty and chronic diseases is complex. The terms primary and secondary frailty have been used to characterize frailty in the absence or presence of chronic diseases. The number of older people with primary frailty is very small. More importantly, development of diseases can precipitate frailty through requiring mobilization of resources by the organism, with consequences for the functional reserves. The development of characteristics of frailty is evident in the advanced stages of many diseases.^26^

In the present study, the most prevalent criterion of frailty syndrome was low physical activity, followed by weakness and exhaustion. The prevalence of the criteria varies according to the population studied, although weakness characterized by low handgrip strength has been the most prevalent criterion in many studies, underscoring the importance of muscle strength in the genesis of the syndrome.^27^^,^^28^^,^^29^

In an investigation on the early manifestations and development of frailty phenotype in women, Xue et al. reported that the incidence of frailty among women who were non-frail at the beginning of the study was 9%.^29^ Despite the heterogeneity observed, weakness was the most common initial manifestation and its occurrence together with slowness and low physical activity preceded exhaustion and weight loss in 76% of the women who were not frail at the beginning of the study.^29^ These findings suggest that weakness can serve as a sign of increased vulnerability in the initial stages of frailty.

The specific cutoff points for the criteria of weakness, slowness and low physical activity that were calculated in this study were lower than those proposed by Fried et al.^2^ These lower values may perhaps be due to the lower values of the anthropometric variables of height and BMI that were used for calculating these parameters.

In a study on frailty, Santos calculated specific cutoff values for his sample regarding the criteria of gait time and handgrip strength.^20^ When these specific cutoff points were used, the frequencies of frail, pre-frail and non-frail subjects were very similar to those observed in the study by Fried et al.^2^ However, there were higher frequencies of frail and pre-frail subjects and a lower frequency of non-frail subjects when the reference values proposed by Fried et al. were used.^2^ This indicates that these cutoff points should be adjusted for the population studied because of the specificity of the anthropometric measurements involved.

The Minnesota Leisure Time Activities Questionnaire, which has already been validated in Brazil,^16^ is a long questionnaire that covers some activities little practiced by elderly Brazilians. This characteristic may have influenced the very low cutoff points detected for this criterion. However, it is important to recognize that the elderly population studied here presented low physical activity levels and that changing this condition is an important goal to be achieved.

Women had worse performance regarding the criteria involving physical effort, i.e. they were slower and had lower energy consumption and lower grip strength in this study. However, because of the lower cutoff points for women than for men regarding these criteria, this difference did not affect the women’s syndrome scores. The only criterion that was significantly more present among women was fatigue.

It is of fundamental importance to recognize frailty syndrome in elderly populations in view of these individuals’ high susceptibility to stressful events. There may be the possibility of halting or even reversing the progression of the syndrome. Although the results from the present study cannot be applied to populations with different characteristics, they may be useful for making estimates for use in medium-sized urban populations and may be of help in drawing up public policies.

The main limitation of this study was its cross-sectional nature, which did not allow any temporal relationship between the variables to be established. On the other hand, cross-sectional studies are important in order to show the burden of frailty in the population and the factors associated with such cases, as done in the present investigation. Studies with this design are subject to survival bias, which could lead to underestimation of the associations observed.

Another limitation that can be mentioned was our decision to exclude patients who were already known to be dependent. This may have influenced the frailty rate obtained. However, this was a decision based on our interest in evaluating frail and potentially vulnerable individuals among independent elderly subjects, since these individuals are not identified through conventional evaluation.

## CONCLUSION

The percentage of frailty syndrome detected in the population of the present study, which was independent for basic activities of daily living, was similar to that observed in developed countries. Frailty was associated with more advanced age, previous stroke and presence of diabetes, neoplasia and urinary and fecal incontinence. Frail elderly individuals attended more visits to doctors and had a higher chance of hospitalization over the past year than did the pre-frail and non-frail groups. The cutoff points detected for weakness, slowness and low physical activity were lower than those proposed by Fried et al., thus indicating that these cutoff points should be adjusted for the population studied. The present study provides data to support public health strategies for early detection of pre-frailty and frailty in Brazilian cities, with a focus on appropriate interventions for prevention and reversal of this syndrome, thereby reducing complications, hospitalizations and mortality.
